# A Reclusive Foreign Body in the Airway: A Case Report and a Literature Review

**DOI:** 10.1155/2013/347325

**Published:** 2013-11-07

**Authors:** Ajay Philip, V. Rajan Sundaresan, Philip George, Satyabrata Dash, Regi Thomas, Anand Job, V. K. Anand

**Affiliations:** Department of Otorhinolaryngology, Head and Neck Surgery, Christian Medical College, Vellore, Tamil Nadu 632004, India

## Abstract

A foreign body in the larynx is an airway emergency that requires urgent evaluation and treatment. Irregular foreign bodies tend to orient in a sagittal plane and may produce only partial obstruction, allowing adequate air movement, hence making them undetectable for a long period of time. We report a case of a laryngotracheal foreign body that remained reclusive for 9 years.

## 1. Introduction

Foreign bodies in the airway are a dire emergency and are a challenge to the otolaryngologist. They require prompt medical attention and rapid airway access. They occur less frequently in adults [1, 2].

Children are the common victims, with the highest incidence being in patients below 15 years [3] of which majority fall in 1–3 age group and of which 25% are below 1 year. The male to female ratio of tracheobronchial foreign bodies varies from 2 : 1 [4, 5] to 3 : 2 [6].

The true incidence may be illusive, and the sole reason is that most symptoms are nonspecific. A history of aspiration may be obtained in patients who present with acute symptoms, but individuals with chronic foreign bodies vaguely remember one [7, 8].

## 2. Case Report

**Figure 1:**
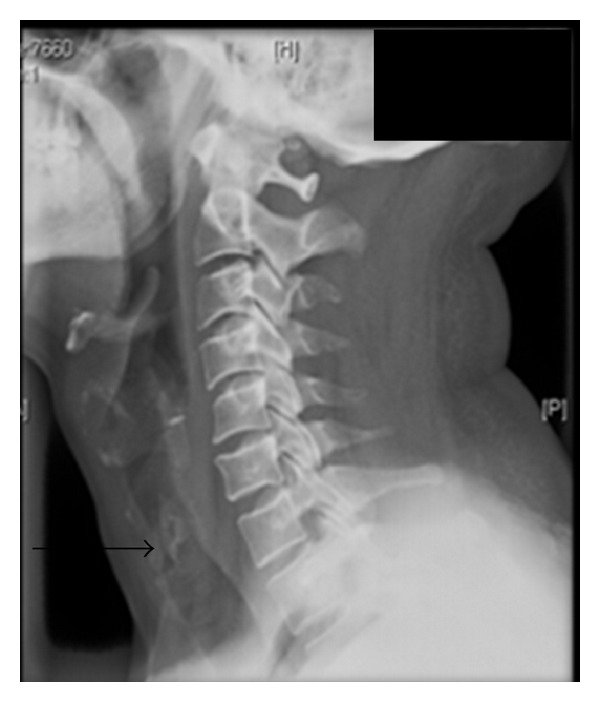
X-ray neck lateral view—radiopaque foreign body at C6-C7 level.

A 39-year-old Indian male presented to our outpatient department with a 9-year history of intermittent odynophagia and hoarseness, associated with noisy breathing. He recalled that his aforementioned symptoms began in a certain day after work; however, he did not seek medical attention. He presented to us 9 years later with mild biphasic stridor and indirect laryngoscopy revealed a subglottic proliferative growth compromising the tracheal luminal airway. X-ray neck lateral view revealed a subglottic narrowing at C6-C7 level (Figure 1) (black arrow).

**Figure 2:**
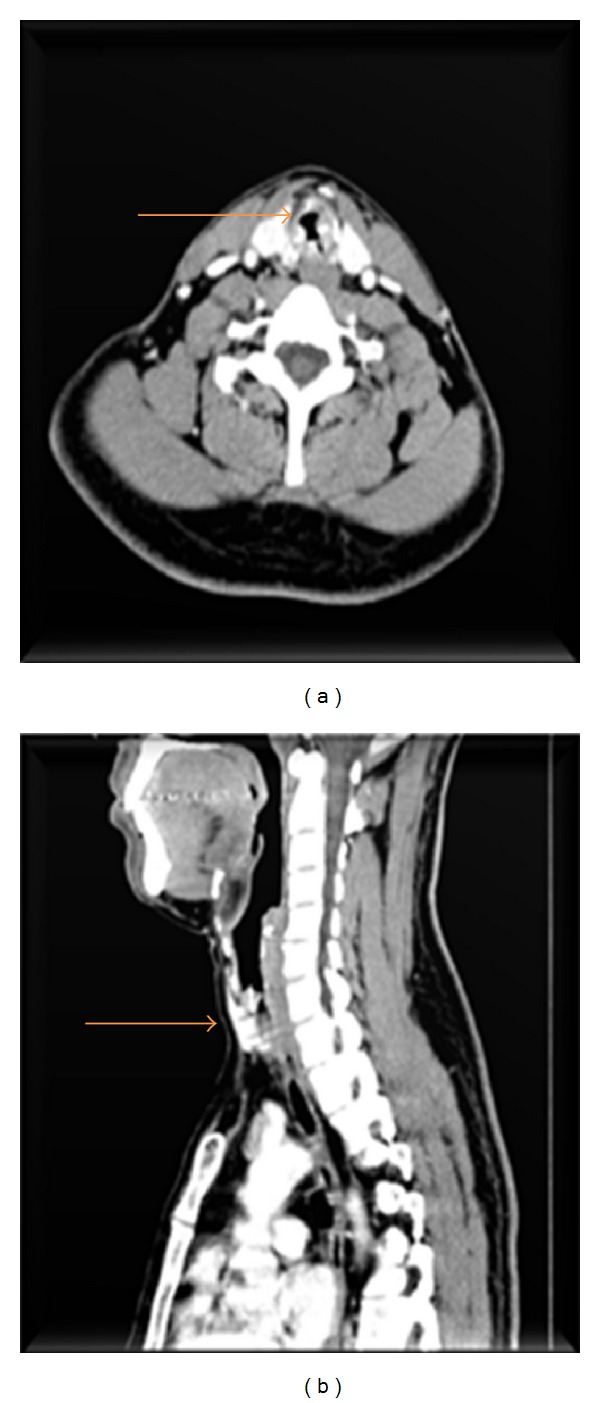
Circumferential thickening in the subglottic region.

Computed tomography of the neck showed a circumferential wall thickening involving the subglottic region and adjoining trachea causing mild luminal narrowing for a segment measuring approximately 27 mm (Figure 2) (orange arrow).

**Figure 3:**
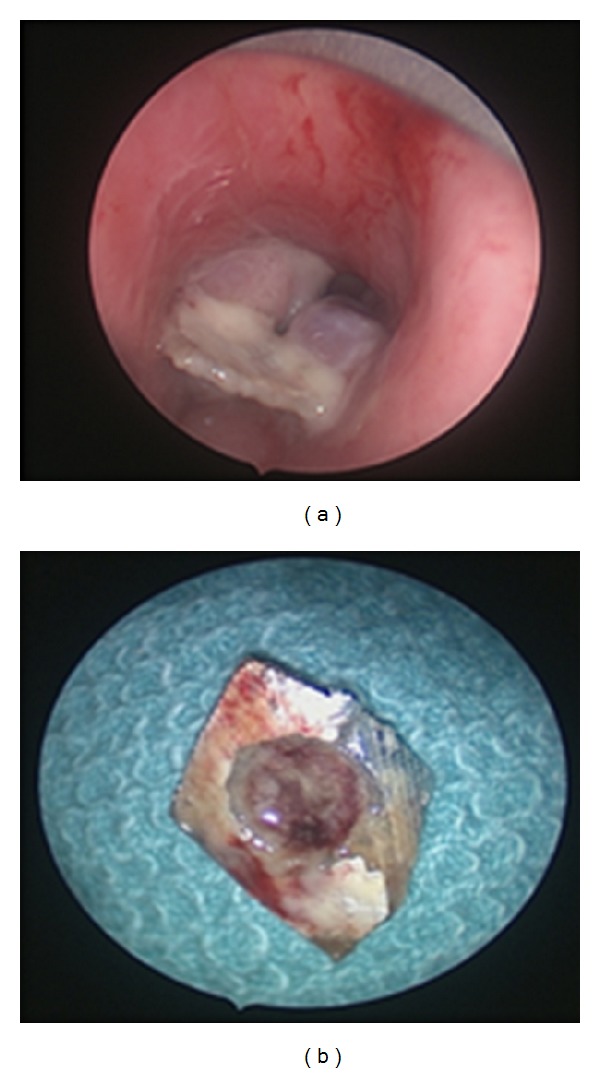
Tablet foil with surrounding granulation tissue.

A working diagnosis of subglottic growth/idiopathic subglottic stenosis was made. The patient was tracheostomized prior to examination under anesthesia in view of a compromised airway. A zero degree telescopic assessment of the larynx was done and a single tablet foil was noted at the level of the first and the second tracheal ring surrounded by thick granulation tissue (Figure 3).

The foil was removed and the adjacent granulation tissue was excised by cold steel excision. Histopathology of the granulation tissue revealed fibrocollagenous tissue covered with acanthotic stratified squamous epithelium with ulceration. He was started on budesonide inhalers postoperatively and was discharged on an 8-size portex cuffed tracheostomy tube. His first postoperative followup a week later showed subglottic granulations obscuring less than 50 percent of tracheal lumen. His tracheostomy tube was downsized to metal Chevalier Jackson tube size 24. A repeat rigid telescopy of the larynx a week later showed airway improvement by about 80 percent. He was successfully decannulated and remained symptom free. He was evaluated 6 weeks later where a repeat rigid telescopy of the larynx showed a normal airway lumen.

## 3. Discussion

The larynx performs an effective sphincteric action to protect the lower airways and it is unusual for a foreign body to get aspirated than to be swallowed. Laryngotracheal foreign bodies are seen more in children, and the common age group is below 15 years [9–12].

The first case of foreign body removal from the trachea was reported by Gustav Killian on March 30, 1897 [13]. In the early days, foreign body removals from the airway were mainly performed by cardiothoracic surgeons and the rigid bronchoscope was frequently utilized for this purpose. Failure to remove the foreign object by the rigid scope was followed by thoracotomy or if necessary bronchotomy. The advent of flexible endoscopy first introduced by Shigeto Ikeda in 1968 revolutionized the care of these patients [14].

Tracheobronchial region is reported to be more involved in children than adults. Due to the nonspecificity of symptoms, the true incidence may be misleading. A history of aspiration may be obtained in patients who present with acute symptoms. Individuals with a chronic history give history of aspiration in 3.4% [15]. The common symptoms are cough, fever, hemoptysis, and dyspnea [15]. Limper et al. found that, in his retrospective study of 60 individuals, 94% presented with cough. McGuirt et al. in a study including 88 patients reported cough and fever in 28% and 17%, respectively, and wheeze in 28% individuals. They reported reduced breath sounds in 47% of his patients [16]. In our patient, biphasic stridor was the chief sign; his air entry was equal bilaterally and had conducted sounds heard during both inspiration and expiration. The longest duration of a chronic foreign body in the airway was reported by Weisberg and Schwartz in 1987 where they reported a chicken breast bone lodged in the bronchus intermedius for 12 years which was later retrieved by bronchotomy [17].

The literature identifies organic materials as the culprit in most cases and varies with local custom. It includes many materials including vegetable matter, watermelons, and bones [15, 18, 19].

In our patient it was a metal foil which remained reclusive for 9 years. The value of radiological tests is invaluable, though X-rays have a high incidence of false negative when done immediately; chest X-rays are frequently used in the assessment of patients with respiratory complaints [20].

Diagnosis would be more obvious in patients with radiopaque foreign bodies (FB). However, radiolucent foreign bodies may pose a problem as they are often missed. In a retrospective analysis, Lufti noted 6.6% demonstrated radiopaque FBs in chest radiographs. Other radiographic abnormalities in the order of frequency included unilateral emphysema (32.2%), atelectasis (12.9%), and infiltration (10.1%) [21]. These radiographic signs, when present, should alert the attending physician to the possibility of aspirated FB, and further specific investigations are warranted. Loo et al. reported 72% of patients having FB impaction on chest radiographs [20].

This was also shown by Svedstrom et al. who reported 67.7% positive chest X-rays in bronchoscopically proven tracheobronchial FB (sensitivity of 67% and specificity of 68%) [22]. Mu et al. reviewed 343 children with proven tracheobronchial FB and found that 62.7% had positive X-ray findings [23].

Rigid bronchoscopy was a valuable tool in tracheobronchial foreign body removal for many years, till the advent of flexible fibreoptic scope. It has a larger working channel permitting use of a variety of instruments at a time and gives an advantage in maneuverability of instruments in comparison to the flexible scopes. It has an edge over the flexible scopes if the foreign body is deeply embedded in granulations as in our case. The flexible scopes can be performed under local anesthesia with few risks and complications, and it allows the exact site of lodgment to be determined. Smaller airways can also be examined by this technique. They are particularly useful in distally lodged foreign bodies [20].

In our patient, we used a microlaryngoscopy and a rigid O degree endoscope to visualize and extract the foreign body. Surrounding granulation tissue was removed by cold steel.

Complete airway obstruction resulting from a foreign body is an absolute emergency. Vegetative and nonvegetative objects (e.g., toys and balloons) commonly lodge in the larynx and trachea. As with laryngeal foreign bodies, edema can progress to complete obstruction. Increased public awareness and the widespread use of the Heimlich maneuver have greatly dropped the mortality of acute obstruction. Prompt recognition of a person in acute airway distress reduces mortality significantly. Back blows or abdominal thrusts in individuals with only partial obstructions could lead to complete obstruction and are not recommended. Patients with tracheal foreign bodies typically do not have hoarseness; Jackson and Jackson described three signs associated with tracheal foreign bodies: (1) “asthmatoid wheeze,” (2) the “audible slap” produced from foreign body contact with the trachea, and (3) the “palpable thud” over the trachea [24].

Surgical management involves direct laryngoscopy, visualization of the foreign body, and passage of a bronchoscope. Preferred bronchoscopes with a rod-lens telescope are the Doesel-Huzly bronchoscopes (Karl Storz). Age-appropriate sizes minimize laryngeal edema. For airway foreign bodies, two bronchoscopes are prepared so that if one fails, another is immediately available. Rigid bronchoscopy is the preferred method for removal of foreign bodies lodged in the airways, but some studies found that flexible bronchoscopy can also achieve a high success rate [25].

The newer optical grabbing forceps available are integrated telescopes which can be passed through the most rigid ventilating bronchoscopes (size of 3.5 and above). This enables the operator to grasp an object such as a peanut under direct vision. Pneumonia and atelectasis are the most common complications after bronchial foreign body removal. Patients usually respond to intravenous antibiotics and chest physiotherapy. Bleeding can occur due to granulation tissue or erosion into a major vessel. An airway tear can cause pneumothorax and pneumomediastinum.

## 4. Conclusion

Air way foreign bodies mostly present as emergencies, but there are also a few cases where they remain reclusive. High index of suspicion with adequate radiological and endoscopic evaluation is a must before embarking on a management protocol.
